# Influence of Na_2_SO_4_ Produced from Phosphogypsum Conversions on the Basic Properties of Building Gypsum

**DOI:** 10.3390/ma18010158

**Published:** 2025-01-02

**Authors:** Danutė Vaičiukynienė, Jūratė Mockienė, Dalia Nizevičienė, Ignas Ramanauskas

**Affiliations:** 1 Faculty of Architecture and Civil Engineering, Kaunas University of Technology, Studentų St. 48, 51367 Kaunas, Lithuania; danute.vaiciukyniene@ktu.lt (D.V.); ignas.ramanauskas@ktu.lt (I.R.); 2 Faculty of Industrial Engineering and Technology, Lietuvos Inzinerijos Kolegija Higher Education Institution, 50155 Kaunas, Lithuania; jurate.mockiene@lik.tech; 3 Faculty of Electrical and Electronics Engineering, Kaunas University of Technology, Studentų St. 48, 51367 Kaunas, Lithuania

**Keywords:** chemical phosphogypsum conversion, building gypsum, Na_2_SO_4_ additive

## Abstract

This study comprises two distinct but interrelated parts. The first part involves optimizing the conditions for the conversion of phosphogypsum to a Ca(OH)_2_ and Na_2_SO_4_ solution. The second part focuses on enhancing the mechanical properties of gypsum through the use of a sodium sulphate additive derived from the conversion of phosphogypsum. An ultrasonic disperser was employed to accelerate the reaction between phosphogypsum and a sodium hydroxide solution. The mean dispersion time was found to be 0.2, 0.5, 1.0, and 2.0 min. The resulting product was a solution of calcium hydroxide and sodium sulfate. The impact of varying quantities of Na_2_SO_4_ on the compressive strength and density of building gypsum samples was investigated. An increase in the quantity of sodium sulphate from 0.2% to 2% resulted in a notable rise in the density of the building gypsum samples, from 1127 kg/m^3^ in the reference sample to 1264 kg/m^3^ in the sample containing 2% sodium sulphate. Therefore, in all instances, the utilization of the Na_2_SO_4_ additive in the gypsum samples resulted in elevated compressive strengths (4.8–8.6 MPa) in comparison to the reference sample devoid of this additive (1.6 MPa).

## 1. Introduction

Various types of waste are generated due to rapid industrialization and urbanization, which pose serious environmental problems. The goal of the circular economy is to decrease resource consumption and increase waste recycling. Phosphogypsum is a byproduct of industrial processes that is not often recycled. This substance is created by converting natural phosphate rock into phosphoric acid. Approximately 5 tons of phosphogypsum are formed for every ton of produced phosphoric acid, resulting in an annual accumulation of 100–280 million tons [1]. Typically, this residual material is disposed of in landfills without processing or treatment. Landfills are typically situated near factories, taking up significant space and harming the environment. Phosphogypsum contains high levels of harmful impurities, including sulfates, phosphates, fluorides, radionuclides, heavy metals, and trace elements, which can cause environmental damage. Up to 15% of the world’s phosphogypsum production is utilized for construction, soil improvement, and Portland cement production. The accumulation of phosphogypsum harms the environment and wastes land resources. One way to use phosphogypsum efficiently is through recycling.

Scientists have conducted extensive research on the recycling and utilization of phosphogypsum in the construction sector, yet the amount of phosphogypsum that is recycled remains minimal. Phosphogypsum recycling can reduce pollution and produce building materials. The process of disposing of phosphogypsum can aid in addressing protection issues in production zones. According to several researchers, adding phosphogypsum to Portland cement is recommended. Singh and colleagues [2] outlined a method for enhancing phosphogypsum quality by purifying an aqueous citric acid solution. This process makes it ideal for manufacturing cement and gypsum plaster. Water-soluble compounds such as citrates, aluminum, and ferrates are produced through this process. Purified phosphogypsum contains fewer impurities (phosphate, fluoride, and organic matter) than untreated phosphogypsum treated with a citric acid solution. Materials made using purified phosphogypsum show similar strength to those made with pure mineral gypsum, including Portland cement and slag cement. According to Ennaciri et al. [3], the recommended method for processing phosphogypsum involves sieving it to 200 μm and treating it with sulfuric acid to eliminate any impurities that cannot be dissolved. The results of this process indicate that, when phosphogypsum is treated with 20% or 50% sulfuric acid at 60 °C for 2 h, it becomes a suitable material for producing plaster and cement.

Taher, M.A. et al. [4,5] have suggested using heat treatment for phosphogypsum. In their study, the substance was heated at temperatures ranging from 200 to 1000 °C in increments of 200 degrees. The study found that heating phosphogypsum reduces impurities like phosphates, fluorides, and organic matter, making it appropriate for Portland cement manufacturing. One way to use phosphogypsum in building materials is by creating composite systems. Degirmenci et al. [6] investigated the potential of combining phosphogypsum, fly ash, and lime in construction. The production of binder utilized heat-treated phosphogypsum as a primary raw material. It has been discovered that the binder produced can be utilized to manufacture bricks and blocks used for interior walls. In a different study [7], researchers explored bricks made from fly ash, lime, and phosphogypsum as an alternative to conventional clay bricks. These bricks are lighter, more durable, and strong enough for construction in aggressive environments. One possible use for phosphogypsum is in creating building materials like construction aggregates. In their study, Foxworthy et al. used phosphogypsum and slag to produce coarse aggregate [8]. This type of aggregate is added to Portland cement concrete, commonly used in highway construction. The coarse aggregate used in this concrete has suitable mechanical properties for highway pavements with Portland cement. Ghafoori et al. [9] found that phosphogypsum has potential as a concrete filler in construction materials. In cement mixtures containing phosphogypsum, calcium sulfate impurities do not negatively impact the product’s long-term properties.

Furthermore, the chemical modification of phosphogypsum is feasible through its conversion into other valuable chemical compounds, thereby expanding the potential applications of this material. The newly created chemical products can be employed in a multitude of industrial sectors, thereby assisting in the resolution of environmental concerns associated with the utilization of phosphogypsum. In a previous study, Ennaciri et al. [10] presented a method for converting phosphogypsum waste into calcium carbonate and lithium sulphate. The initial materials employed were phosphogypsum and lithium carbonate. Following chemical modification, the resulting products can be employed in the manufacturing of lithium batteries (LiSO_4_) and the cement industry (CaCO_3_). The optimal conditions for chemical modification were found to be a reaction time of 1.5 h at room temperature and stoichiometric proportions of phosphogypsum and Li_2_CO_3_. In a further study [11], the optimal conditions for the conversion of phosphogypsum were refined. The objective was to enhance the conversion of phosphogypsum to CaCO_3_ through the utilization of Na_2_CO_3_ as a starting material. Based on variance analysis (ANOVA), the optimal conversion conditions were identified as a concentration of Na_2_CO_3_ solution at 30%, a reaction time of 10 min, and a solid/liquid ratio of 1:2.

In another study [12,13], high-purity Ca(OH)_2_ and Na_2_SO_4_ were obtained from Moroccan phosphogypsum by treating it with a caustic soda solution. Phosphogypsum conversion was performed at room temperature using stoichiometric amounts of starting materials. The study results were compared with the results of the conversion of building plaster. When Moroccan phosphogypsum is exposed to a potassium alkali solution, K_2_SO_4_ and CaCO_3_ are formed, which can be used in the agricultural, chemical, and construction industries [14]. Ennaciri et al. [15] exposed phosphogypsum to a K_2_CO_3_ solution, and, in this case, K_2_SO_4_ and CaCO_3_ were formed as reaction products. Optimal phosphogypsum conversion was obtained at 80 °C using stoichiometric amounts of phosphogypsum and K_2_CO_3_.

Ennaciri et al. [16] proved that the reaction of phosphogypsum with NaF produced CaF_2_ and Na_2_SO_4_, which can be used in the glass and metallurgical industries. In this case, optimal conversion was achieved after 90 min at room temperature using stoichiometric ratios of the starting materials. Zemni et al. [17] used another combination of starting materials for phosphogypsum conversion. Phosphogypsum was made in a sodium silicate solution, and, after conversion, sodium sulfate and calcium silicate were formed. Agayr et al. [18] proved that Na_2_CO_3_ and NH_4_HCO_3_ aqueous solutions can convert phosphogypsum successfully. The optimal conversion rate of PG into Na_2_SO_4_ was found to be 96–98%, and (NH_4_)_2_SO_4_ was 96%.

Mohammed et al. [19] investigated the conversion of phosphogypsum by exposing it to solutions of sodium salts such as NaOH and Na_2_CO_3_. Thus, Na_2_SO_4_ (Equations (1) and (2)) is formed when phosphogypsum is exposed to sodium salts.

CaSO_4_·2H_2_O + 2NaOH → Ca(OH)_2_ + Na_2_SO_4_ + 2H_2_O. (1)

CaSO_4_·2H_2_O + Na_2_CO_3_ → CaCO_3_ + Na_2_SO_4_ + 2H_2_O. (2)

Thus, this work aimed to chemically modify phosphogypsum into calcium hydroxide and sodium sulfate and use the resulting sodium sulfate as an additive to improve the physical–mechanical properties of building gypsum.

## 2. Methods and Materials

### 2.1. Experimental Techniques

The elemental composition of the initial materials was determined via X-ray fluorescence (XRF) analysis, using a Bruker X-ray S8 Tiger WD spectrometer featuring a rhodium (Rh) tube. The anode voltage was 60 kV, and the electric current intensity was 160 mA. The powder samples were processed in a helium atmosphere. The results were analyzed using the SPECTRA Plus QUANT EXPRESS method.

The mineral composition (XRD) was investigated with the X-ray diffractometer DRON—7, with a Bragg–Brentano geometry. The device applied Ni-filtered CuKα radiation and a graphite monochromator, using 30 kV voltage and 20 mA emission current. The peaks corresponding to the mineral compounds were identified using the Oxford Cryosystems Crystallographica Search-Match 3.1 software and the PDF-2 database.

A microstructural analysis was performed with a scanning electron microscope, (SEM) Hitachi S-3400N Type II, with image resolutions of secondary electrons in a high vacuum of ≥3 nm (when the potential difference is 30 kV), and ≥10 nm (when it is 3 kV). The applied acceleration voltages for different enhancements were 5 and 15 kV.

In order to determine the mechanical properties of gypsum and phosphogypsum, cubes of 2 × 2 × 2 cm were formed from paste of a normal consistency. The water/cement ratio and the setting time of the mixture (normal consistency) were determined in accordance with EN 196-3 [20]. The cubes were compressed with the press ELE AutoTest. The compressive strength of phosphogypsum specimens was measured according to EN 196-1 [21] and the modulus of elasticity according to ISO 6784 (1982) [22].

To determine the hydration degree of the hemihydrate PG, the loss-on-ignition method (LOI) was employed. Firstly, 2 g of the material was calcined at 400 °C for 1.5 h. After that, the LOI value was calculated as the relative difference between the initial and final mass of the specimen. The Gibbs free energy of the main chemical reactions involved in the conversion of phosphogypsum to Na_2_SO_4_ was calculated using HSC Chemistry 10 software.

### 2.2. Initial Materials

**Figure 1:**
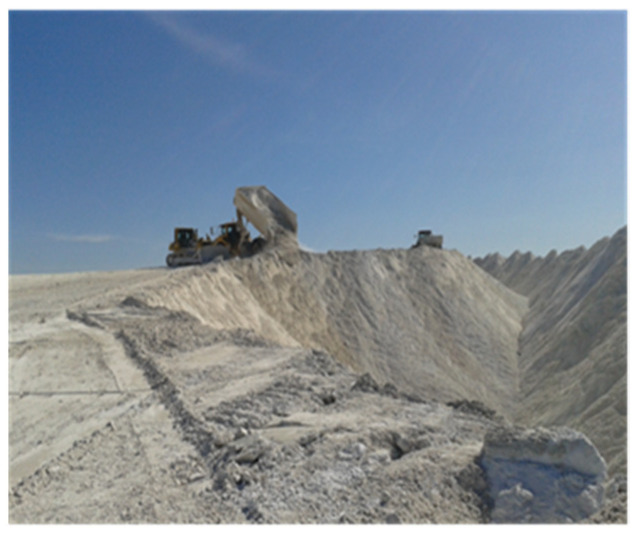
Phosphogypsum mountains situated in the Kėdainiai District of Lithuania.

Waste-hemihydrate phosphogypsum (α-CaSO_4_·0.5H_2_O) is produced by UAB LIFOSA (Lithuania) during the production of extractive phosphoric acid. It is formed by the reaction of sulfuric acid with apatite obtained from the Kovdor area on the Kola Peninsula. Unfortunately, most phosphogypsum (CaSO_4_·2H_2_O) is stored near factories in mountainous areas and remains unprocessed (Figure 1). Therefore, there is an urgent need to find ways to recycle and use this material.

The phosphogypsum was taken from the waste mountains and dried at 40 ± 5 °C. Table 1 provides the typical chemical composition of the phosphogypsum. In this material, SO_3_ and CaO dominate, and the sum of these oxides is 91.38%. Other oxides, such as P_2_O_5_, SiO_2_, Al_2_O_3_, and others, do not exceed 1.37%. Building gypsum mainly consists of SO_3_ and CaO.

**Figure 2:**
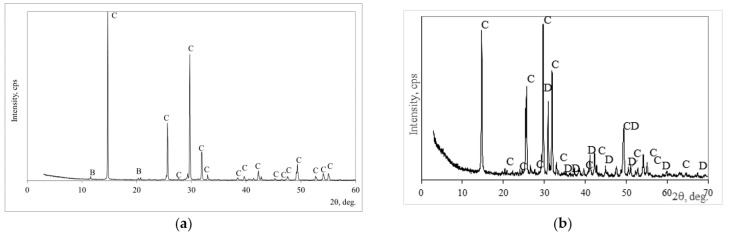
The X-ray diffraction patterns of phosphogypsum (**a**) and building gypsum (**b**). Notes: C is basanite CaSO_4_·0.5H_2_O (33–310), B is brushite CaPO_3_·(OH)·2H_2_O (11–293) and D is dolomite (84–1208).

The mineral composition of the phosphogypsum was determined according to X-ray analysis, and it is presented in Figure 2a. The calcium sulfate semi-hydrate Ca(SO_4_)·0.5(H_2_O) dominated in phosphogypsum with the impurities of brucite CaPO_3_ (OH)_2_·H_2_O. A similar mineral composition had building gypsum (Figure 2b): semi-hydrated Ca(SO_4_)·0.5(H_2_O) dominated, and dolomite with anhydrite was detected as an impurity.

**Figure 3:**
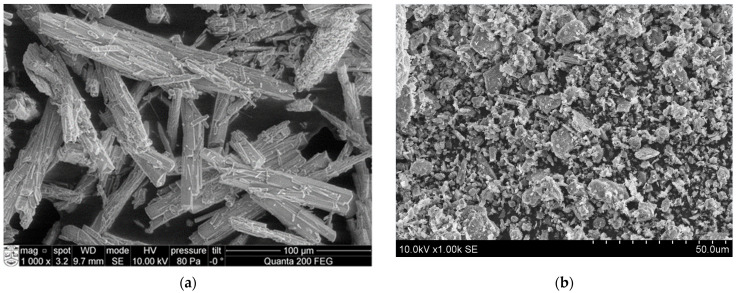
The microstructures of phosphogypsum (**a**) and building gypsum (**b**).

Scanning electron microscopy (SEM) analysis was used to determine the microstructure of the initial materials such as phosphogypsum and building gypsum. In Figure 3, it is evident that phosphogypsum consists of distinct prismatic and needle-shaped crystals, with an average length measuring between 100 and 200 µm and a diameter measuring between 15 and 20 µm. In Figure 3b, it can be seen that building gypsum is composed of finer particles compared to phosphogypsum particles [23,24,25,26].

Sodium hydroxide (NaOH) in granule form was used as a laboratory reagent material. The white granules used in this study were sourced from Russia.

Latvian-made building gypsum is produced using German KNAUF technology from natural gypsum raw materials. Table 1 details the average chemical composition, and the mineral composition was determined through the XRD method (Figure 3b).

### 2.3. Chemical Conversion of Phosphogypsum

During the experimental studies, a chemical conversion process of phosphogypsum was carried out, during which a solution of sodium sulfate (Na_2_SO_4_) and solid calcium hydroxide (Ca(OH)_2_) was formed.

The optimal reaction conditions involved mixing 1.5 M phosphogypsum with a stoichiometric amount of 3 M sodium hydroxide solution. The reaction was conducted at a temperature of 20 °C with stirring at 500 rpm for 30 min [13].

Several experiments were carried out to evaluate the reaction of phosphogypsum with the NaOH solution. The aim was to find the optimal conditions, such as the duration of the conversion reaction and the concentration of the products obtained. This study focused on using 1.5 M phosphogypsum and 3.0 M NaOH concentrations. The experiment did not include excess NaOH, and the reaction between phosphogypsum and the sodium alkali solution followed Reaction (3).

CaSO_4_·0.5 H_2_O + 1.5 H_2_O → CaSO_4_·2 H_2_O
CaSO_4_·2 H_2_O + 2 NaOH → Ca (OH)_2_+ Na_2_SO_4_ + 2 H_2_O (3)

Thermodynamic calculations were evaluated prior to the experiments. When water was added to semi-hydrated calcium sulphate, it was hydrated to dihydrate calcium sulphate with a Gibbs free energy of ΔG = −37.397 kJ. This reaction was exothermic (energy release). The second chemical reaction had a Gibbs free energy of ΔG = −76.810 kJ and was also exothermic because the Gibbs free energy was ΔG < 0. The second reaction was more negative than the semi-hydrated calcium sulphate hydration reaction.

An ultrasonic dispergator was used to accelerate the reaction, and average processing times of 0.2, 0.5, 1.0, and 2.0 min were selected. The amounts of initial materials and processing conditions are provided in Table 2.

**Figure 4:**
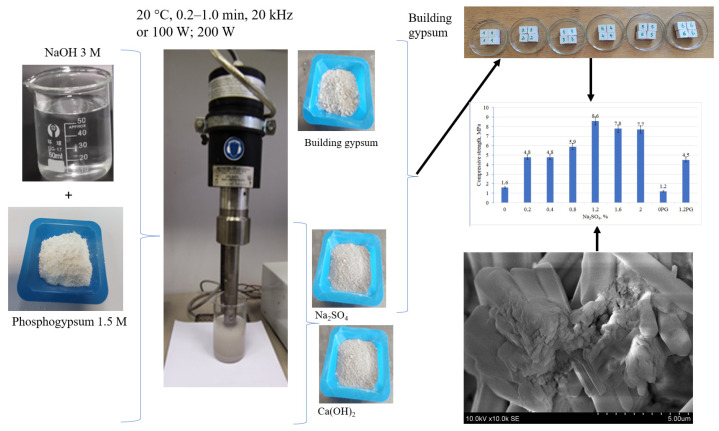
Scheme of influence of Na_2_SO_4_ addition on the basic properties of building gypsum.

The mechanical properties of the gypsum samples were enhanced through the addition of Na_2_SO_4_, specifically the compressive strength. The Na_2_SO_4_ was derived from the conversion of phosphogypsum with sodium hydroxide. The microstructure of the hydrated building gypsum samples underwent alterations (SEM, scanning electron microscopy), which subsequently influenced the strength of the samples (Figure 4).

## 3. Results and Discussion

### 3.1. Optimizing the Conditions for the Conversion of Phosphogypsum to Ca(OH)_2_ and Na_2_SO_4_

**Figure 5:**
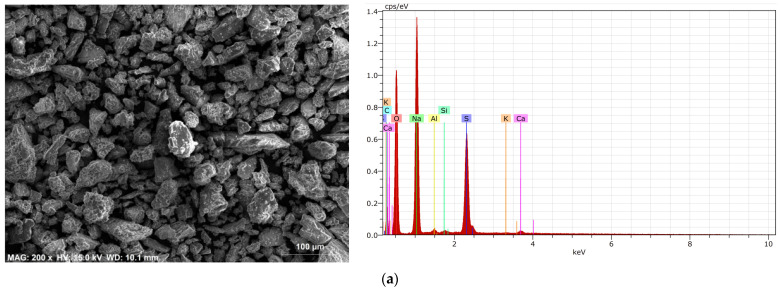

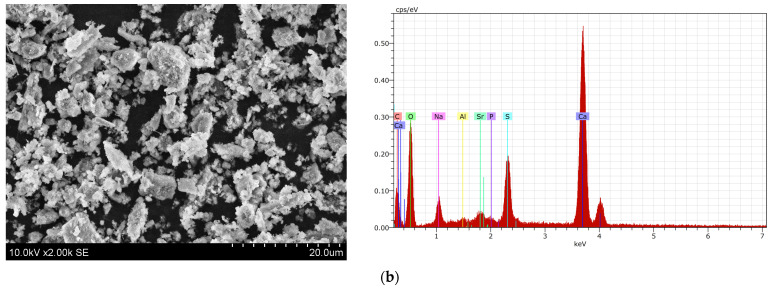
The microstructure and elemental composition of sodium sulphate (Na_2_SO_4_) (**a**) and calcium hydroxide (Ca(OH)_2_) (**b**) obtained by conversion from phosphogypsum.

Following the completion of the reaction between phosphogypsum and sodium hydroxide (Reaction (3)), the resulting solid products were filtered using a Buchner filter. The solid Ca(OH)_2_ precipitate was collected on the filter. Given that Na_2_SO_4_ dissolves in water, it remained in the aqueous solution from which the calcium hydroxide precipitate was separated. To form sodium sulphate in solid form, the water was evaporated, resulting in the formation of a solid compound. The elemental composition of the ground sodium sulphate was determined and is presented in Figure 5.

**Figure 6:**
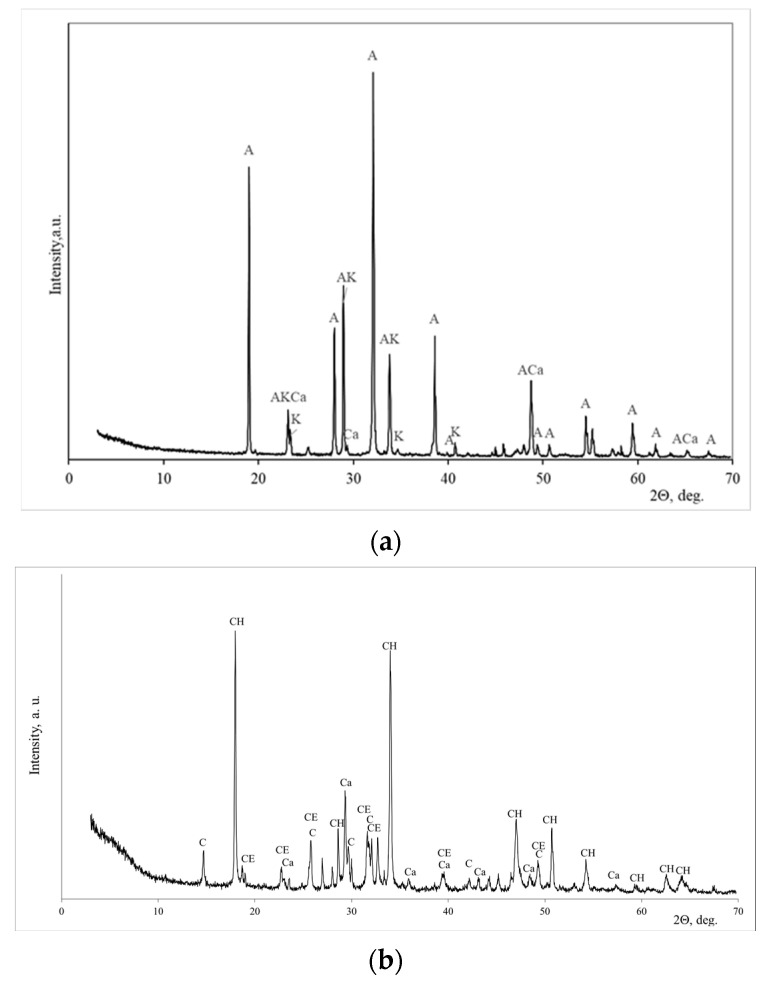
The X-ray diffraction pattern of sodium sulphate (Na_2_SO_4_) (**a**) and calcium hydroxide (**b**). Notes: A is thenardite Na_2_SO_4_ (74–2036), K is burkeite Na_6_(SO_4_) (24–1134), Ca is calcite CaCO_3_ (1–837), CH is calcium hydroxide Ca(COH)_2_ (84–1265), C is basanite CaSO_4_·0.5H_2_O (33–310), and Ce is cesanite Ca_1.31_Na_4.32_(OH)_94_(SO_4_)_3_ (75–1691).

The SEM images show that the microstructure of both samples consists of sharp edges and particles of different sizes. The EDS analysis (Figure 5a) shows that the sodium sulphate samples are predominantly sodium and sulfur, while the calcium hydroxide is predominantly calcium (Figure 5b). The mineral composition was determined by XRD, and the sodium sulphate sample was dominated by thenardite, with small amounts of burkeite and calcite (Figure 6a). The mineral composition of the second phosphogypsum conversion product, Ca(OH)_2_, was found to be calcium hydroxide with calcium carbonate, with minor amounts of basanite and cesanite (Figure 6b).

**Figure 7:**
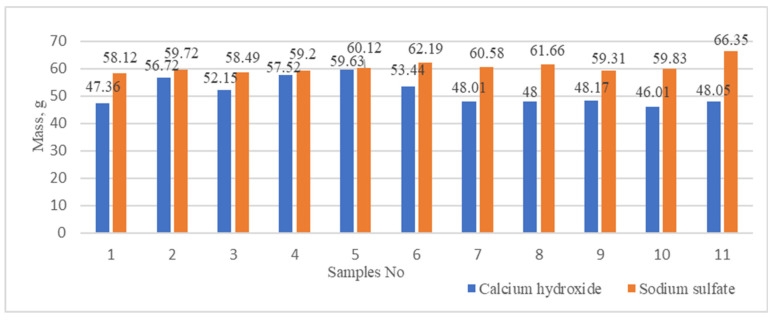
Dependence of the mass of calcium hydroxide (Ca(OH)_2_) and sodium sulfate (Na_2_SO_4_) on the mixing method and ultrasonic dispergation parameters of the suspensions.

The mass of new formations produced during the conversion of phosphogypsum to calcium hydroxide and sodium sulfate was determined (Figure 7). The use of ultrasonic dispersion at a lower power setting (100 W) yielded an average of 55.89 g of calcium hydroxide, with a maximum of 59.63 g (Figure 7). Nevertheless, an increase in the intensity of ultrasonic dispersion to 200 W resulted in a slight reduction in the average amount of calcium hydroxide, which was approximately 48.17 g, with an average value of 47.65 g (Figure 7).

**Figure 8:**
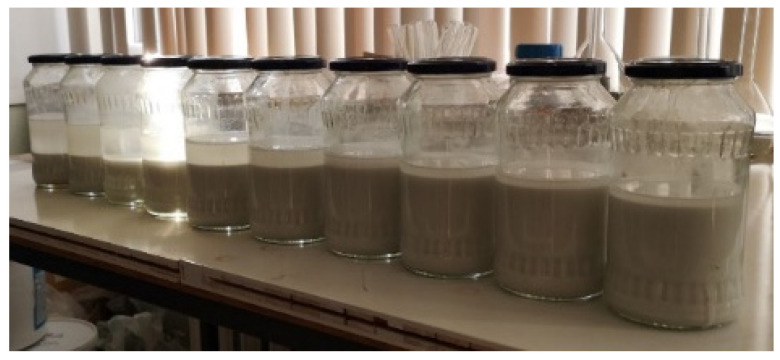
Photographs of phosphogypsum and caustic soda suspensions after ultrasonic treatment.

The duration of ultrasonication of the suspension was increased from 0.2 to 2.0 min, resulting in the enhanced formation of calcium hydroxide and the improved dispersibility of the solid phase. Furthermore, an increase in the ultrasonic treatment power led to a notable enhancement in the dispersity of the solid material phase, as illustrated in Figure 8. This photograph depicts a clear reduction in the size of solid material particles, accompanied by an increase in the volume of solid materials.

Similar results were obtained by Mahbubul et al. [27] by increasing the dispersion of Al_2_O_3_ nanoparticles in H_2_O using ultrasonic dispergation. The best results in terms of particle dispersion and cluster size reduction were achieved with an ideal duration of about 3–5 h. In this study, the particle size distribution analysis showed that the cluster size decreased with an increasing ultrasonic duration.

**Figure 9:**
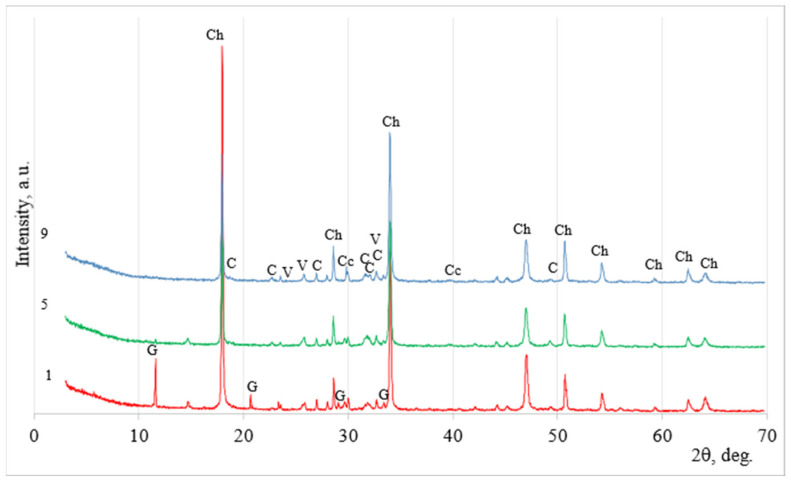
Mineral composition of phosphogypsum sample precipitation affected by sodium alkali according to XRD analysis. Notes: Ch is calcium hydroxide Ca(OH)_2_ (84–1271), G is gypsum CaSO_4_·2H_2_O (70–982), V is vaterite CaCO_3_ (72–506), and Ca is calcite CaCO_3_ (72–1651).

The examination of the solid portion revealed that the main mineral was calcium hydroxide Ca(OH)_2_ ‘Ch’, as shown in Figure 9 by X-ray analysis. However, sample No. 1 (which had not been subjected to ultrasonic dispersion) still contained a significant amount of gypsum CaSO_4_-2H_2_O ‘G’, as indicated in the graph. The gypsum peaks disappeared when ultrasonic dispersion was used. The rapid carbonation of calcium hydroxide resulted in the formation of calcite ‘Cc’ and vaterite ‘V’.

### 3.2. The Influence of Sodium Sulfate Additive on the Properties of Building Gypsum Samples

Building gypsum is an air binder made from hemihydrate calcium sulfate, obtained by partially dehydrating dihydrate calcium sulfate gypsum rocks or waste gypsum materials.

Table 3 illustrates that the initial and final setting times of the reference sample (devoid of any additives) were 17 and 20 min, respectively. However, when the Na_2_SO_4_ content was 1.2%, the initial and finale setting times were reduced to 4/5 min. The situation was similar with semi-hydrated phosphogypsum. The addition of Na_2_SO_4_ (1.2%) shortened the setting time, and, in this case, a retardant should be used. Phosphogypsum without additives (8* sample) had a shorter settings time than the gypsum samples (5/6 min). This can be explained by the fact that CaSO_4_2H_2_O remained in the binder and acted as crystallization centers [28].

The gypsum samples consisted of gypsum and water. The water-to-gypsum ratio was 0.4, determined according to a normal paste consistency. Sodium sulphate was also included, ranging from 0% (in the reference sample) to 2% depending on the amount of gypsum used.

**Figure 10:**
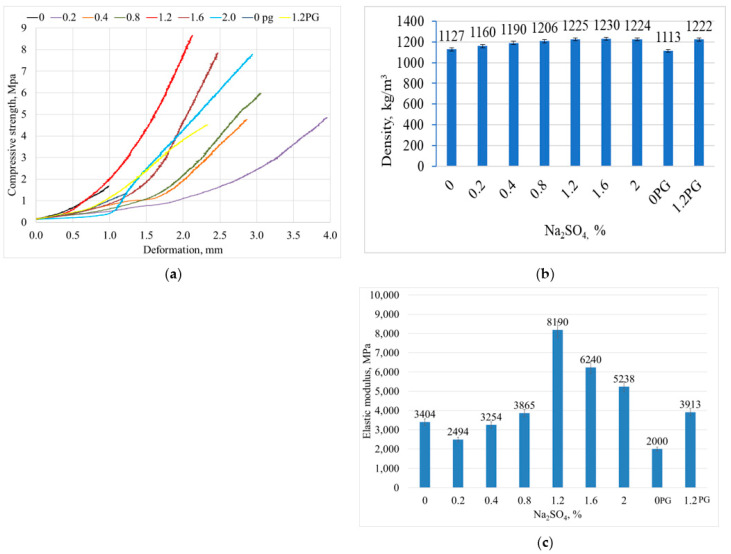
The influence of the amount of Na_2_SO_4_ additive on the compressive stress–strain response (**a**) density (**b**) and elastic modulus (**c**) of the gypsum samples.

As illustrated in Figure 10a, the maximum compressive strength (8.6 MPa) of the building gypsum sample was achieved with this amount of additives. Conversely, when the amount of additives was increased to 2%, the compressive strength was observed to decrease by 10.5% (7.7 MPa). Building gypsum is made from a common sedimentary mineral. Gypsum products are widely used in building materials and have a high fire resistance. The compressive strength of building gypsum (β type) can reach 4–6 MPa (EN 13279-2:2014) [29], while high-strength gypsum (α type) can have a strength of up to 15–40 MPa. The maximum compressive strength of phosphogypsum was lower than that of building gypsum and was only 4.5 MPa with the addition of Na_2_SO_4_ [30].

Thus, according to this experimental study, the addition of sodium sulphate accelerated hydration and led to the formation of more short needle-like interlocking crystals, resulting in higher strength values of the gypsum and phosphogypsum than the reference sample. Zhi et al. [31] reports that alkali metal sulphates accelerate the hydration of gypsum systems. Sodium sulphate at optimal levels had a positive effect on the increase in strength, accompanied by the formation of a denser structure.

As shown in Figure 10a, the results indicate that the maximum compressive strength of the dry gypsum samples was 8.6 MPa after seven days of hydration. This value was more than five times higher than that of the reference sample.

The addition of Na_2_SO_4_ in small amounts up to 0.4% slightly reduced the modulus of elasticity for the gypsum samples, but, when the additive was present in amounts higher than 0.4%, the modulus of elasticity was higher than that of the reference sample without the additive in every case (Figure 10c). For the phosphogypsum samples, the addition of Na_2_SO_4_ at more than 0.4% increased the elastic modulus. In the case of the phosphogypsum samples, the addition of Na_2_SO_4_ at more than 0.4% increased the modulus of elasticity.

**Figure 11:**
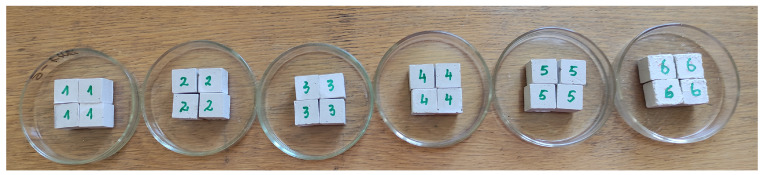
Picture of gypsum samples with different Na_2_SO_4_ additions.

Moreover, an increase in the sodium sulphate concentration from 0.2% to 2% resulted in a notable rise in the density of the gypsum samples, from 1127 kg/m^3^ in the reference sample to 1264 kg/m^3^ in the sample with 2% sodium sulphate (Figure 10b). Therefore, in all instances, the utilization of the sodium sulphate additive in the gypsum samples resulted in augmented compressive strengths. However, with a higher amount of additive, an excess of sodium sulphate was formed, and some of it accumulated on the surface of the samples, as shown in Figure 11.

**Figure 12:**
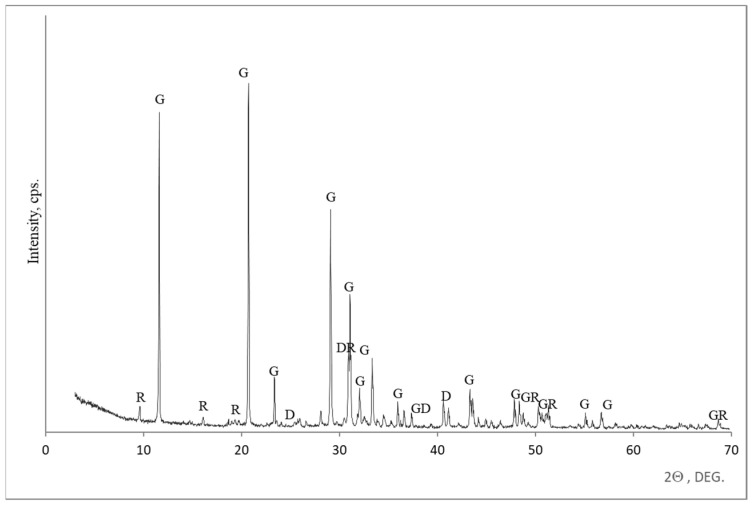
Mineral composition of building gypsum samples according to XRD analysis. Notes: G is gypsum CaSO_4_ 2 H_2_O (33–311), D is dolomite Ca Mg (CO_3_)_2_ (84–1208), and R is hydroglauberite Na_10_ Ca_3_ (SO_3_)_8_ ·6 H_2_O (24–1071).

On the surface of building gypsum sample No. 6 (Figure 11), irregularities and some solid particles were detected. In order to clarify the mineral composition of those traces, an XRD analysis was performed (Figure 12). The XRD analysis showed that these traces contained dihydrated calcium sulphate, which was predominantly present with a small amount of hydroglauberite.

**Figure 13:**
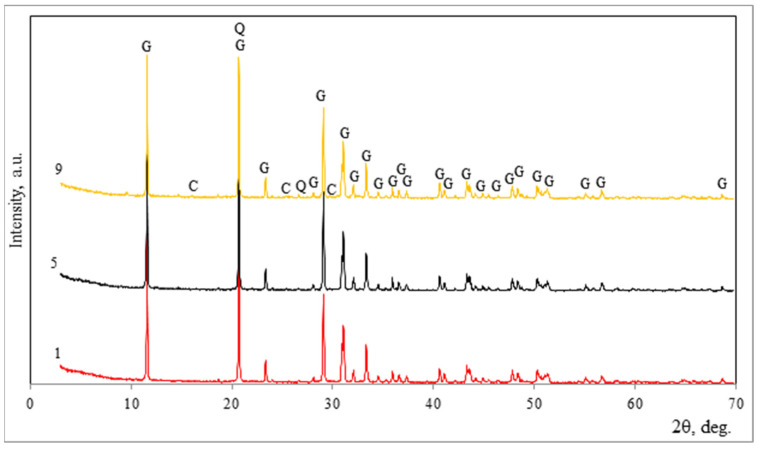
XRD analysis of mineral composition for building gypsum samples. Notes: G is gypsum CaSO_4_·2 H_2_O (33–311), C is basanite CaSO_4_·0.5 H_2_O (33–310), and Q is quartz SiO_2_ (83–2465).

This study examined mineral composition both with and without sodium sulphate. The results indicate that calcium sulphate dihydrate was the main mineral present in all samples, as shown in Figure 13. Additionally, a small amount of quartz was detected. This trace element might have been a result of gypsum’s origin from natural calcium sulphate dihydrate.

**Figure 14:**
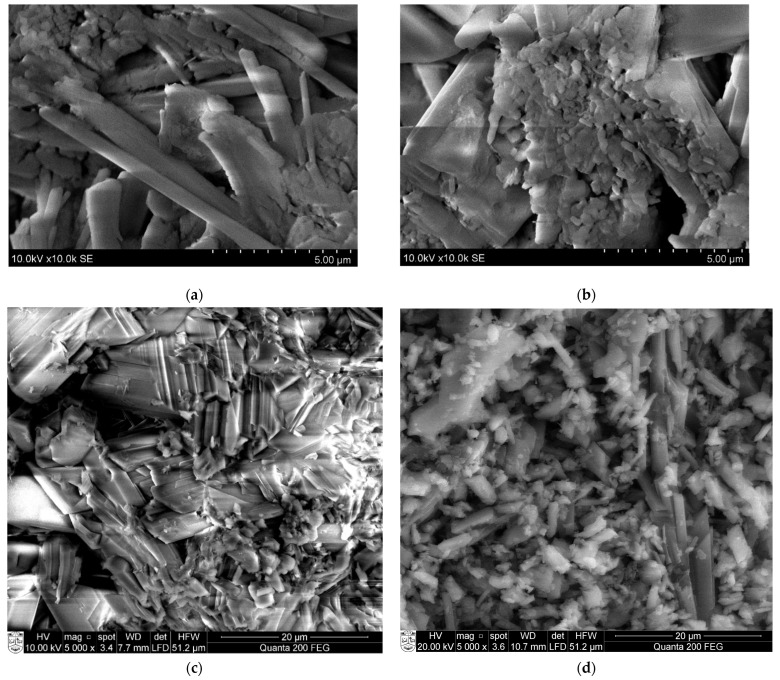
Microstructure of hydrated building gypsum (**a**,**b**) and phosphogypsum (**c**,**d**) samples: (**a**,**c**) sample without additive; and (**b**,**d**) sample with the addition of sodium sulphate (Na_2_SO_4_).

The compressive strength of the building gypsum samples was related to its microstructure (see Figure 14). A study conducted by Moghadam et al. [32] found that the addition of Na_2_SO_4_ (sodium sulfate) to gypsum sped up the hydration process and resulted in the formation of shorter, needle-shaped interconnected crystals. This led to a stronger gypsum matrix. The researchers used SEM images to observe that the size of the needle-shaped gypsum crystals decreased with the addition of sodium sulfate. The average length of the needle-shaped crystals, calculated from 20 crystals based on their SEM image, was around 13 μm, 10 μm, and 6 μm, respectively, in decreasing order of sodium sulfate in the gypsum samples. The microstructure of gypsum, in the presence of sodium sulphate addition, was needle-shaped with interlaced and smaller crystals. This could enhance the mechanical strength of the samples. With the addition of sodium sulphate, nucleation can occur rapidly, leading to the growth of smaller crystals and ultimately forming a denser structure. This study suggests that the final strength of gypsum is related to the formation of gypsum crystals and the bond between them. The SEM pictures confirm that gypsum strength is affected by the formation of gypsum crystals and joints. The effect of Na_2_SO_4_ addition on the microstructure of phosphogypsum samples was similar. The microstructure of the additive-free samples was dominated by prismatic conglomerates. However, the addition of 1.2% sodium sulphate made the microstructure finer and more compact. In this case, finer prismatic conglomerates predominated. The finer structure had a positive effect on strength development.

## 4. Conclusions

The chemical conversion of phosphogypsum was carried out in two ways: whole suspension, i.e., without sonication, and sonication. After the conversion of the phosphogypsum in an unstirred suspension, some unreacted dihydrated gypsum was found in the solid reaction products. In the chemical conversion of phosphogypsum by sonication, the reaction products were dominated by calcium hydroxide as a solid. Insignificant amounts of calcite, vaterite, and cesanite were also found in the solid products. The best condition for the chemical conversion of phosphogypsum was 1.5 M phosphogypsum and 3.0 M sodium hydroxide solution with ultrasonic dispergation (at 20kHz and 100 W).

The effect of the Na_2_SO_4_ additive on the properties of building gypsum and phosphogypsum was investigated. The addition of 0.2 to 2% sodium sulphate increased the strength of the gypsum and phosphogypsum samples in all cases. The highest compressive strength (8.6 MPa) and elastic modulus (8190 MPa) were found for the samples with 1.2% sodium sulfate addition. The mechanical strength could be attributed to changes in the microstructure, i.e., with a larger number and finer needle-shaped connected crystals. In this study, it was found that the addition of sodium sulphate had a significant effect on the strength properties of gypsum and phosphogypsum and that the matrix was more compact and denser.

## Figures and Tables

**Table 1:** Chemical composition of phosphogypsum and building gypsum, in %.

Materials	CaO	MgO	SO_3_	P_2_O_5_	SiO_2_	Al_2_O_3_	K_2_O	F	Other	LOI *
PG	40.12	0.03	51.26	1.10	0.41	0.07	0.01	0.08	0.35	7.25
Building gypsum	37.1	3.60	44.3	-	4.97	1.63	0.714	-	0.79	6.90

* LOI—loss on ignition.

**Table 2:** Initial material mixtures and processing conditions of conversions of phosphogypsum to Ca(OH)_2_ and Na_2_SO_4_.

No.	PG, (mol/L)	NaOH, (mol/L)	Duration, min	Methods of Mixing Suspensions
1	1.5	3.0	30	Unmixed
2	1.5	3.0	0.2	Ultrasonic dispergation at 20 kHz and 100 W
3	1.5	3.0	0.5	Ultrasonic dispergation at 20 kHz and 100 W
4	1.5	3.0	1	Ultrasonic dispergation at 20 kHz and 100 W
5	1.5	3.0	1.5	Ultrasonic dispergation at 20 kHz and 100 W
6	1.5	3.0	2	Ultrasonic dispergation at 20 kHz and 100 W
7	1.5	3.0	0.2	Ultrasonic dispergation at 20 kHz and 200 W
8	1.5	3.0	0.5	Ultrasonic dispergation at 20 kHz and 200 W
9	1.5	3.0	1	Ultrasonic dispergation at 20 kHz and 200 W
10	1.5	3.0	1.5	Ultrasonic dispergation at 20 kHz and 200 W
11	1.5	3.0	2	Ultrasonic dispergation at 20 kHz and 200 W

**Table 3:** The impact of Na_2_SO_4_ on the setting times of gypsum. Water-to-gypsum ratio, W/G = 0.4.

Sample No.	Na_2_SO_4_, %	Setting Time, min	
		Initial	Final
1	0	17	20
2	0.2	10	12
3	0.4	8	9
4	0.8	5	6
5	1.2	4	5
6	1.6, difficult to dissolve in water	4	5
7	2, does not dissolve in water	3	4
8 *	0	5	6
9 *	1.2	2	4

*—Semi-hydrated phosphogypsum was used as a binder.

## Data Availability

Data are contained within the article.
